# Weak genetic structure despite strong genomic signal in lesser sandeel in the North Sea

**DOI:** 10.1111/eva.12875

**Published:** 2019-11-01

**Authors:** Belén Jiménez‐Mena, Alan Le Moan, Asbjørn Christensen, Mikael van Deurs, Henrik Mosegaard, Jakob Hemmer‐Hansen, Dorte Bekkevold

**Affiliations:** ^1^ Section for Marine Living Resources National Institute of Aquatic Resources Technical University of Denmark Silkeborg Denmark

**Keywords:** *Ammodytes marinus*, fisheries management, genetic structure, lesser sandeel, population genetics, stock management, structural variation

## Abstract

Sandeels are an ecologically important group of fishes; they are a key part of the food chain serving as food for marine mammals, seabirds and fish. Sandeels are further targeted by a large industrial fishery, which has led to concern about ecosystem effects. In the North Sea, the lesser sandeel *Ammodytes marinus* is by far the most prevalent species of sandeel in the fishery. Management of sandeel in the North Sea plus the Kattegat is currently divided into seven geographical areas, based on subtle differences in demography, population dynamics and results from simulations of larval dispersal. However, little is known about the underlying genetic population structure. In this study, we used 2,522 SNPs derived from restriction site‐associated DNA sequencing (RADseq) typed in 429 fish representing four main sandeel management areas. Our main results showed (a) a lack of a clear spatially defined genetic structure across the majority of genetic markers and (b) the existence of a group of at least 13 SNPs under strong linkage disequilibrium which together separate North Sea sandeel into three haplotype clusters, suggestive of one or more structural variants in the genome. Analyses of the spatial distribution of these putative structural variants suggest at least partial reproductive isolation of sandeel in the western management area along the Scottish coast, supporting a separate management. Our results highlight the importance of the application of a large number of markers to be able to detect weak patterns of differentiation. This study contributes to increasing the genetic knowledge of this important exploited species, and results can be used to improve our understanding of population dynamics and stock structure.

## 1 INTRODUCTION

Marine fishes are often characterized by high fecundity, large effective population sizes and high dispersal potential leading to weak patterns of genetic differentiation (Ward, Woodwark, & Skibinski, 1994). As a consequence, studies relying on few genetic markers may lack the statistical power to identify local populations and to assess connectivity among spatially defined stock units. Fisheries management requires information on the distribution and vital rates of biological units within specific management areas, as failure to recognize biological units with different demographics may lead to overfishing and ultimately depletion of less productive population units (Kerr et al., 2016). In spite of the generally low differentiation observed across the genome in a number of marine fishes, sequencing approaches now allow for analyses of large numbers of DNA markers, resulting in greatly enhanced power for identifying genomic regions exhibiting genetic structure (Bernatchez et al., 2017; Nielsen et al., 2012). Such signatures may be associated with local adaptation or reveal traces of cryptic population structure obscured by gene flow across most of the genome (Duranton et al., 2018; Gagnaire et al., 2015; Nielsen et al., 2012).

In this study, we used double‐digest restriction site‐associated DNA (ddRAD) sequencing to develop and analyse genetic markers in samples of lesser sandeel, *Ammodytes marinus* (L.) from the North Sea. Collectively, five species of sandeel with partly overlapping distributions can be found in the North Sea (ICES, 2017); they are key components of the food web, serving as food for fish, seabirds and marine mammals (Furness, 2007). Lesser sandeel is the most abundant fish species in the North Sea and is the core target of an industrial fishery for fishmeal mainly in the North Sea (ICES, 2017). *A. marinus* is a short‐lived benthic species feeding in the pelagic zone over the bottom of sandy gravel banks, otherwise burrowing into the substrate for up to 8 months a year (Wright, Jensen, & Tuck, 2000). The species is expected to be nonmigratory and larvae to drift by ocean currents settling mostly within a regional range of 50–100 km around local spawning sites (Christensen, Jensen, Mosegaard, St. John, & Schrum, 2008; Jensen, Rindorf, Wright, & Mosegaard, 2011; Wright, Christensen, Régnier, Rindorf, & van Deurs, 2019). These characteristics have led to the suggestion that *A. marinus* may display adaptation to local conditions (van Deurs, Hartvig, & Steffensen, 2011; Wright et al., 2019). The species is considered to follow a “boom‐and‐bust” dynamic, characterized by large population size fluctuations and the occasional survival of very large numbers of young fish that are able to sustain a large fishery for a couple of years, followed by low productivity periods (van Deurs, van Hal, Tomczak, Jónasdóttir, & Dolmer, 2009; Henriksen et al., 2018; Lindegren et al., 2018). Time series analysis suggests that the most important population regulation mechanism is either inter‐cohort competition or cannibalism of larvae by 1‐year‐old conspecifics, which gives a clear 2‐year cycle in stock‐recruitment time series (Arnott, Ruxton, & Poloczanska, 2002; van Deurs et al., 2009). North Sea sandeel has undergone large temporal variations in population sizes, and catches have varied between 75,405 and 1,217,839 tons (average = 585,704 tons) over the past 30 years (ICES, 2018). Debates on how to identify biologically meaningful stock units have prompted investigation into population structure and connectivity based on inference from data on spatial recruitment patterns, morphological markers and larval drift patterns based on hydrographic modelling (see Wright et al., 2019). Apart from one study examining three allozyme markers and showing a lack of differentiation among samples from the North Sea and Norwegian Sea (Nævdal & Thorkildsen, 2002), no study has hitherto examined genetic population structure in lesser sandeel.

In this study, we aimed to (a) characterize the population structure of lesser sandeel in the North Sea using population genomic data and (b) assess whether the management areas currently implemented for the species in the North Sea are in line with observed genetic variability.

## 2 METHODS

### 2.1 Sample collection

**Figure 1:**
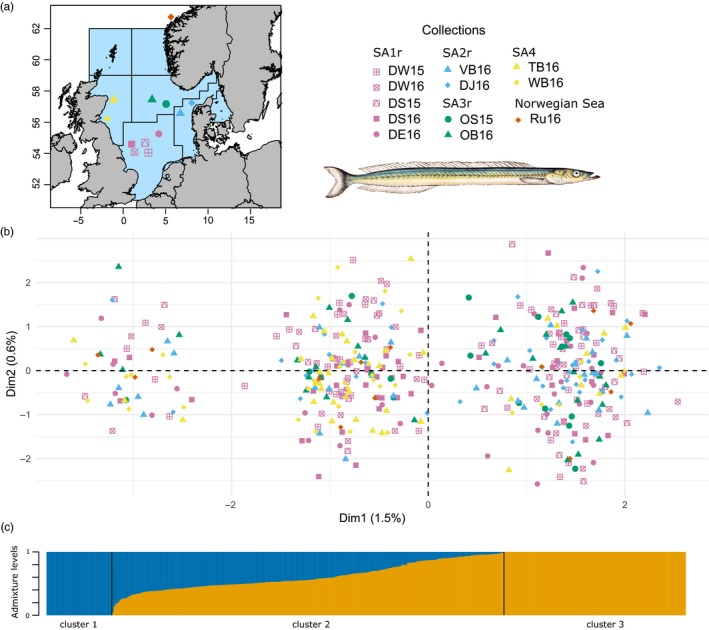
(a) Map of collection locations. Each colour and shape represent a different sampling collection and management area, respectively, and the grey lines represent the sandeel management areas 1–7 (ICES, 2017). Illustration of *Ammodytes marinus* by Gervais and Boulart (1877) obtained from Wikimedia Commons. (b) Principal component analysis (PCA) of individuals for all loci (2,522 SNPs), with shapes and colours representing the collections and management areas, respectively, as in (a). Individuals are projected along the PC1 and PC2 axes, and distributed into cluster 1 (left), cluster 2 (middle) and cluster 3 (right). Percentage of variation explained by each axis is also included. (c) Levels of ADMIXTURE of each individual, ordered according to the *Q* values from ADMIXTURE results for all loci with a model of 2 lineages (*K* = 2). Each colour represents the proportion of ADMIXTURE in relation to each cluster in the PCA

*Ammodytes marinus* were collected from 11 sandbank spawning locations during the Danish and Scottish dredge surveys (ICES, 2017) in November–December 2015 and 2016 (Table S1). Collections represented four current North Sea sandeel management areas (SA1r, SA2r, SA3r, SA4, Figure 1a) that are applied by ICES as separate units for advice on fisheries management (ICES, 2018). An additional collection from the north‐western coast of Norway (>500 km away) was included for comparison. Samples were collected in October–December which is close to the spawning season (in December–January) and consisted of a mixture of size classes representing both juvenile and adult life stages (Figure S1). Two collections in 2016 were taken on Dogger Bank West (DW) and in proximity to Dogger Bank South (DS), close to sandbanks also sampled in 2015 (Table S1). For some of the analysis, these samples in DW and DS in 2015 were considered as temporal replicates within locations and were removed to avoid pseudoreplication.

### 2.2 DNA extraction and library preparation

DNA was extracted from 60 to 70 individuals per collection site, and between 40 and 44 individuals per collection were randomly selected for library preparation (Peterson, Weber, Kay, Fisher, & Hoekstra, 2012). For each selected individual, DNA was standardized at 20 ng per µl and processed with two restriction enzymes, Pst1 and Msp1, with a rare and frequent cutting site, respectively. Sixty individuals were then randomly pooled per library in equimolar proportion and were size‐selected on an agarose gel in order to obtain an insert size range from 350 to 450 bp. After a PCR amplification phase (14 cycles), the libraries were purified using AMPure beads. The quality of each library was controlled by using the high‐sensitivity DNA reagent on a Bioanalyzer 2100 (Agilent Technologies). In total, nine libraries were sequenced in paired end (2 × 100 bp) using nine lanes of a HiSeq 4000 at an external sequencing centre. To avoid potential sequencing bias, sequencing libraries consisted of a mix of individuals from different collections.

### 2.3 Bioinformatics and data filtering

Illumina libraries were demultiplexed using *process_radtags* in STACKS v1.46 (Catchen, Hohenlohe, Bassham, Amores, & Cresko, 2013). A first filtering step was done at this stage, where reads with low‐quality scores (‐q, below a phred score of 10) or with uncalled bases (‐c) were discarded. Reads were trimmed for the 7‐bp barcodes. We enabled the option to retain barcodes and RADtags (‐r). Paired‐end reads with more than ten overlapping bases were merged using FLASH (Magoc & Salzberg, 2011) with default parameters. After visually checking quality in FastQC (Andrews, 2010), all reads were trimmed to 174 bp. To do this, we followed two different procedures for the merged and nonmerged reads. For the merged reads, we discarded reads shorter than 174 bp and trimmed the longer ones to 174 bp using Trimmomatic (Bolger, Lohse, & Usadel, 2014). For the reads that did not overlap, we reverse complemented the R2 sequence and trimmed the beginning (13 bp) and the end (6 bp) after visual exploration of sequence quality in FastQC. We then concatenated the two paired‐end reads using a Python script adapted from Settepani et al. (2017). At the end, both merged and concatenated reads (174 bp) were pooled together into individual FASTQ files. The FASTQ files were processed using the de novo pipeline from STACKS v1.46. This pipeline was set with an optimized set of parameter values (*m* = 5, *M* = 5 and *n* = 6), after a careful exploration of the parameters using an ad hoc simulation script (data not shown), following optimization recommendations from Paris, Stevens, and Catchen (2017). We obtained a total coverage of reads of 36.5X. Figure S2 shows the number of reads obtained per collection. To call SNPs, we required a locus to be sequenced in at least 80% of the individuals across collections. Loci showing heterozygosity >0.8 were removed to avoid including paralogous sequences. Only one SNP per tag and SNPs with a minor allele frequency (MAF) above 1% were retained. We excluded individuals with less than 750,000 reads and more than 10% missing data. Departure from Hardy–Weinberg equilibrium (HWE) was tested for each collection using the function *gl.report.hwe* as implemented in the R package “dartR” (Gruber, Unmack, Berry, & Georges, 2018), which includes Bonferroni correction for multiple testing. Figure S3 summarizes the main pipeline and filtering steps used to obtain the SNP data set for this study.

### 2.4 Analysis of genetic diversity and population structure

Using the function *gl.basic.stats* implemented in the R package “dartR,” we estimated overall basic population genetics statistics per locus, such as the overall diversity (H_t_) and the *F*_ST_ corrected for the number of individuals (*F*_ST_’). This function makes use of the functionalities of the R package “hierfstat” (Goudet, 2005). We also performed a neutrality test using BayeScan v2.1 (Foll & Gaggiotti, 2008), with parameters *‐n 5000 ‐thin 10 ‐nbp 20 ‐pilot 5000 ‐burn 50000 ‐pr_odds 100*. BayeScan detects selection signatures by using an *F*_ST_ outlier approach. This method identifies loci potentially under selection or linked to sites under selection, as loci showing departure from the expectation under a neutral demographic model. We also used “pcadapt” to explore selective outliers (Luu, Bazin, & Blum, 2017), which were considered those that had a *p*‐value (after Bonferroni correction) lower than the expected false discovery rate (set to 1%). To explore population structure, we performed a principal component analysis (PCA) using the function *dudi.pca* from the R package “adegenet” (Jombart & Ahmed, 2011), after replacing missing data with the mean allele frequencies, using no scaled allele frequencies (scale = FALSE). Then, we ran a discriminant analysis of principal components (DAPC) based on the number of clusters suggested by the function *find.cluster* to describe the structure observed in PCA (Jombart, Devillard, & Balloux, 2010). We analysed the ancestry proportions per sample using ADMIXTURE v1.3.0 (Alexander, Novembre, & Lange, 2009) for k ranging 1–5 based on the Bayesian information criteria (BIC) analysis from the DAPC. We estimated *F*_ST_ (Weir & Cockerman, 1984) between all pairwise collections and tested for significance based on permutation tests using the R packages “StAMPP” (Pembleton, Cogan, & Forster, 2013) and “dartR.”

### 2.5 Analysis of linkage disequilibrium: haplotype groups

We estimated linkage disequilibrium (LD) using the *LD* function from the R package “genetics” (Warnes, Gorjanc, Leisch, & Man, 2013), by calculating the square correlation between alleles of each pair of loci, *r* ^2^ (Hill & Robertson, 1968). There is no reference genome available for *Ammodytes,* preventing inference about physical linkage. We instead took advantage of the graphic network‐based method implemented in LDna analysis (Kemppainen et al., 2015) and identified clusters of SNPs under strong LD. The LD network analysis allowed us to explore potential clusters of SNPs with high LD compared to the rest of the data set, without requiring a reference genome. Following Kemppainen *et al.*’s recommendations, we chose 1% of the SNP data set to be the minimum number of edges for a cluster to be considered an outlier by LDna analysis (|*E*|_min_ = 26), and an intermediate threshold of LD (*ϕ* = 4) to extract the maximum number of loci that are in higher LD with each other. We then used data solely for SNPs that were identified by LDna analysis as being highly linked in a second PCA to define haplotype clusters. To describe the genetic make‐up of each cluster identified in the PCA, we estimated heterozygosity and *F*_ST_ across individuals contained within the three main clusters observed (see below). Heterozygosity for this subset of SNPs was estimated as the number of heterozygous genotypes over the total number of loci. We also blasted flanking sequences for the SNPS identified by LDna analysis using the *blastn* function from ncbi‐blast v.2.6.0+ (parameters: ‐db nr ‐query ‐max_target_seqs 5 –remote) (Camacho et al., 2009).

### 2.6 Analysis of population structure and molecular variance (AMOVA)

To explore correspondence between genetic structure and designated management areas, we calculated pairwise *F*_ST_ among management areas and collections. To assess the proportion of genomic variation distributed within and among management areas, we performed an analysis of molecular variance (AMOVA) (Excoffier, Smouse, & Quattro, 1992) implemented in the R package “poppr” version 2.8.2 (Kamvar, Tabima, & Grünwald, 2014). For this analysis, we grouped spatial samples hierarchically within management areas and excluded the temporal samples DW15 and DS15 (to avoid pseudoreplication), and the Norwegian sample (Ru16) to restrict our analysis to the North Sea management areas. We only included SNPs under strong LD detected by the LDna analysis, as it is recognized that, in populations with high gene flow, focusing on outlier markers can help reveal patterns of differentiation not reflected by neutral markers (Gagnaire et al., 2015). Variation among samples within each collection and within individuals was also evaluated. A randomization test with 1,000 replications was used to assess statistical significance. For the two locations with temporal data (DW and DS), we performed a separate AMOVA to assess temporal differences. Finally, we performed a Hardy–Weinberg equilibrium test of the frequencies of the group of loci identified in LDna analysis as in high LD (*χ* ^2^ = 3.84, 1 degrees of freedom) for both individual collections and management areas, using an ad hoc R script.

### 2.7 Environmental association analysis

To explore potential relationships between genetic structure and local environments, we analysed the association between genotypes and environmental factors associated with sandeel feeding conditions (zooplankton biomass) and ambient temperature conditions. These environmental factors are related to the sandeel population dynamics in the North Sea (Christensen et al., 2008; Lindegren et al., 2018) and could potentially be drivers of local adaptation. A total of four variables were considered: temperature at depth of zooplankton maximum (T_ZMAX_), temperature at sea bottom (T_SBT_), depth of zooplankton maximum (D_ZMAX_) and maximum concentration of zooplankton in the water column (C_ZMAX_). The environmental covariates were extracted from the operational coupled physical–biogeochemical model HBM‐ERGOM set‐up for the North Sea/Baltic Sea area (Berg & Poulsen, 2012; Neumann, 2000; Neumann, Fennel, & Kremp, 2002), in a hindcast spanning the period 2004–2013. Within the model data, the water column at each station is assessed at noon at every day starting June 1st and 60 days forward, corresponding to the early foraging period of settled sandeel, after the drift larvae phase. At each station each day, the zooplankton abundance maximum (where sandeel is presumed to forage) is located by scanning down the water column in the model data set. At the zooplankton abundance maximum, the depth and temperature are recorded. Additionally, water temperature is recorded at the seabed position (where sandeel bury after foraging). We averaged the daily data from each environmental factor to obtain a single value per location per factor. Only SNPs under strong LD were considered in this analysis, following the same reasoning as with the AMOVA. Associations between allele frequencies and environmental co‐variables were tested using *glm* analysis that accounted for geographical position of each sampling site. Each factor was tested in a separate model and compared to the null model with an ANOVA test to determine whether a model in‐ or excluding the factor showed association with allele frequencies.

### 2.8 Hydrographic connectivity analysis

We explored whether the genetic data from the LD group aligned with the spatial location of hydrographic dispersal barriers identified through modelling of relative larval transport probability (Christensen et al., 2008; Wright et al., 2019). To do this, we extracted estimates of the average direct connectivity between pairwise sampling sites. Connectivity indices were calculated using a Lagrangian framework (Christensen, Mariani, & Payne, 2018) coupled offline to the HBM‐ERGOM data set described above. Biological dynamics of sandeel larvae were modelled as in Christensen et al. (2008) using March 20th as larval hatch day, settlement at 40 mm larval length and larval growth as described by model 3 in table 2 of Christensen et al. (2008). To construct a simple representation of transport indices, sandeel foraging habitats were projected onto a 10 × 10 km grid cell (corresponding to the resolution of the hydrodynamic model), and transport indices were computed as the probability of successful transport from one cell to another. Briefly, the model operates by the “release” of batches of larvae in each cell at hatch time, following all larvae by drift simulation, and recording where each released larva ends at settlement time (see Christensen et al., 2008). Since 596 grid cells in the model are occupied by sandeel habitats, the transport indices constitute a 596 × 596 matrix, giving the probability of transport between all (directional) pairs of 10‐km grid cells. This analysis excluded information for the locations Ru16 and DJ16 (the model did not comprise those sites) and the temporal samples from 2015. We performed a Mantel test between the matrix of pairwise connectivity estimates and the pairwise *F*_ST_ between the collections using the function *mantel.rtest* implemented in the R package “ade4.” To account for unidirectional dispersal probabilities between locations, we used the mean probability of dispersal for each pairwise location in the Mantel test, as done in White et al. (2010).

## 3 RESULTS

### 3.1 Genetic variation and population structure

We obtained 2,635 SNPs after running the pipeline implemented in Figure S3. In total, 113 SNPs showed significant departure of HWE for all collections and were excluded from further analysis. The final data set consisted of 2,522 loci genotyped in 429 individuals. Each collection was represented by 13–44 individuals (Table S1), with an average of 2.05% missing data. Average H_t_ across loci and samples was 0.113, and overall differentiation was low (*F*_ST_’ = 0.0004). No outlier loci were detected using BayeScan, while “pcadapt” highlighted 25 (data not shown). The first and second principal components in the PCA accounted respectively for 1.5% and 0.6% of the total inertia and did not reveal any geographically explicit structure (Figure 1b). Lack of geographical structure was also evident in analyses of *F*_ST_ between pairwise collections, with estimates from 0 to 0.0036, including between the geographically isolated sample from the Norwegian Sea and all North Sea samples (below diagonal in Table S2). Only one comparison was significantly different from 0 (WB16 vs. DJ16). Still, the first axis of the PCA revealed three well‐defined genetic clusters (Figure 1b), driven by relatively few loci, as revealed by the loading plot (Figure S4). The three clusters observed in the PCA were corroborated by the DAPC. In DAPC, the first discriminant function grouped genotypes into three nonoverlapping clusters, using 100 axes of principal components that explained 46% of the variation (Figure S5). The ADMIXTURE analysis showed that two lineages were sufficient to describe the structure observed in the PCA (Figure 1c, upper left inset from Figure S5), as the cross‐validation (CV) error increased substantially with *K* > 2. The ancestry coefficient *Q* was strongly correlated to each individual's axis position for PC1 (*r* = .97, *p* < 2.2e‐16). Hence, cluster 2 in the DAPC analysis corresponded to individuals with admixed ancestry, and clusters 1 and 3 represented genotypes that were pure for one or the other lineage (Figure 1c).

### 3.2 Study of the genetic clusters identified in PCA

**Figure 2:**
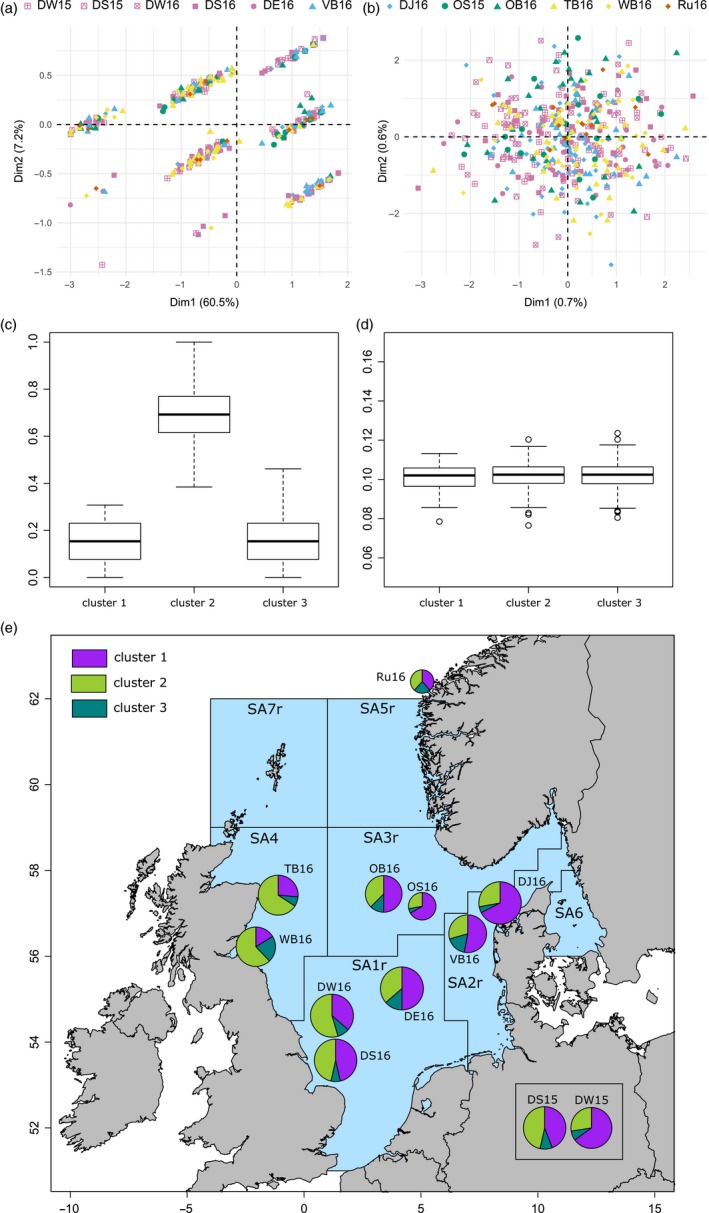
(a) PCA with the 13 SNPs that show strong linkage disequilibrium according to LDna analysis (LD group “49_0.18”). Each shape and colour represent a different sampling collection and management area, respectively, as represented in Figure 1a. (b) PCA excluding the SNPs forming the LD group “49_0.18.” (c) Heterozygosity of the three clusters from PC1 when including the SNPs that formed LD group “49_0.18.” (d) Heterozygosity of the three clusters from PC1 when excluding the SNPs that formed LD group “49_0.18.” Plots in (c) and (d) have a different scale on *y*‐axis. (e) Map of the proportions of individuals within clusters 1, 2 and 3 for each sampling collection. Sandeel management areas 1–7 are indicated (ICES, 2017)

LD among SNPs was low on average (mean = 0.0019, median = 0.0006), but 27 loci (approximately 1%) displayed relatively high values of LD (*r* ^2^ > .5). *LDna analysis* identified a single cluster of SNPs in high LD (cluster “49_0.18” in Figure S6). This cluster contained 13 SNPs with a median LD of 0.29 and a mean of 0.32. All thirteen loci were also among the SNPs loading above the 5% quantile on PC1 in the PCA (Figure S4) and among the 25 outliers detected by “pcadapt.” In agreement with this, a PCA of these 13 SNPs again identified three clusters at PC1 (Figure 2a). Interestingly, for PC2 of this analysis samples were further subdivided into three clusters, although PC2 explained far less variation (7.2%) than PC1 (60.5%). The first two PCs thus identified nine discreet clusters when the 13 loci from the most prominent LD cluster were analysed separately. When we excluded the 13 SNPs, the three clusters disappeared from the PCA (Figure 2b). To describe the genetic make‐up of each cluster identified along the axis that explained more variation (PC1), we estimated heterozygosity and *F*_ST_ for the 13 SNPs exhibiting high LD across individuals contained within three clusters. Individuals showing admixed ancestry (corresponding with cluster 2 in Figures 1b,c and 2a) showed an excess of heterozygous sites across loci, with a mean H_obs_ of 0.71. In comparison, the mean heterozygosity of the two nonadmixed clusters was five times lower, estimated at H_obs_ 0.16 and 0.15, for respectively clusters 1 and 3 (Figure 2c). The global *F*_ST_ for the 13 LD SNPs estimated between the two nonadmixed clusters was high (*F*_ST_ = 0.77). Out of the 13 SNPs, 11 SNPs were fixed, or close to fixation, in at least one of the cluster groups (Figure S7). *F*_ST_ estimated between the admixed and either of the nonadmixed clusters was three times lower (*F*_ST_ cluster 1 vs. 2 = 0.28, *p* = 0; *F*_ST_ cluster 2 vs. 3 = 0.31, *p* = 0). When the 13 SNPs in strong LD were excluded, heterozygosity was similar among the three clusters (range between 0.08 and 0.12, mean of the three clusters = 0.10; Figure 2d), and *F*_ST_ was several orders of magnitude lower (*F*_ST_ clusters 1 and 3 = 0.0032, *p* = 0; *F*_ST_ clusters 2 and 3 = 0.001, *p* = 0; *F*_ST_ clusters 1 and 2 = 0.0003, *p* = .155). The sandeel sequences from eight out of the 13 linked SNPs identified by LDna analysis blasted against genomic regions of fish species (e‐values ranging from 10^–10^ to 10^–30^, Table S3) and 2–4 SNPs blasted to a single chromosome within a species. Five species, mainly marine Perciformes genera, were the most frequently identified among aligning sequences (Table S3).

### 3.3 Assessment of current management areas

Pairwise *F*_ST_ between management areas using the 13 loci under stronger linkage disequilibrium ranged from 0 to 0.06 (Table 1). SA4 showed the highest *F*_ST_ values in comparisons with all other three management areas (all statistically significant). When comparing sampling sites within management areas, *F*_ST_ values ranged from 0 to 0.145 (above diagonal in Table S2). All pairwise *F*_ST_ estimates including WB16 were statistically highly significant, also after correction for multiple testing. WB16 showed the highest *F*_ST_ with DJ16 (*F*_ST_ = 0.145, *p* < .0001), DW15 (*F*_ST_ = 0.112, *p* < .0001) and OS15 (*F*_ST_ = 0.105, *p* < .0001), and the lowest with its neighbouring location TB16 (*F*_ST_ = 0.012, *p* < .05) (Table S2). In the AMOVA, more than 95% of the total variation was partitioned within individuals (Table 2). Variance associated with management areas corresponded to 1.92% (*p* = .02; Table 2). When we excluded the management area SA4 that included WB16, the proportion of variation distributed among management areas decreased to 0.4% (*p* = .22). When substituting the 2016 samples for the 2015 samples for DS and DW in the AMOVA test, the variance associated with management areas was similar to estimates from samples from 2016, but only showed marginal statistical significance (variance = 1.99%, *p* = .07; variance without SA4 = 0%, *p* = .57). When only looking at the samples for where we had temporal data in 2015 and 2016 (DS and DW), the variation estimated between years was 1.73% for DW but was not significant (*p* = .06). The location DS showed no variation between years (variance = 0%, *p* = .85). Individual genotype proportions within both collections and management areas did not deviate from Hardy–Weinberg Equilibrium proportions (Table S7).

**Table 1:** Pairwise *F*_ST_ between North Sea sandeel management areas, based on the 13 most linked SNPs from the LDna analysis (group “49_0.18”)

	SA1	SA2r	SA3r	SA4
SA1	NA	NA	NA	NA
SA2r	0.0095**	NA	NA	NA
SA3r	−0.001	0.0014	NA	NA
SA4	0.0208***	0.0646***	0.0413***	NA

Note

Statistical significance is reported as: *p‐value ≤.05; **p‐value ≤.01; ***p‐value ≤.001.

**Table 2:** Analysis of molecular variance (AMOVA) of *A. marinus* among four management areas, among and within nine collections, and within the 333 individual fish. This analysis is based on the 13 most linked SNPs from the LDna analysis (group “49_0.18”)

	Degrees of freedom	Sum of squares	% of variation	Phi	*p*‐value
Among management areas	3	36.014	1.92	0.02	.02
Among locations within management areas	5	17.86	0.41	0.004	.19
Among samples within locations	324	892.93	2.41	0.025	.20
Within samples	333	873.48	95.26	0.05	.07

Analyses of association between genotypes and environmental factors (Table S4) returned a lack of marked relationships. Of the four environmental factors analysed, only the model containing temperature at the sea bottom (*T*_SBT_) showed a marginally significant correlation with variation in the 13 high‐LD loci (AIC = 55.89; chi‐squared test: *p* = .09; Table 3 and Table S5). Model‐based estimates of drift connectivity between the collection sites are shown in Table S6. The comparison of estimates of dispersion probabilities and genetic divergence indicated a negative correlation between connectivity between sampling sites and *F*_ST_, as expected under isolation by geographical distance. However, the Mantel test was not statistically significant (Mantel observed correlation = −0.17, *p* = .89).

**Table 3:** Summary statistics from the chi‐squared test comparing the model with and without the environmental factor that explains the proportion of the inversion type in each geographical location

Environmental factor	AIC	*p*‐value (*χ* ^2^ test)
Null (Long*lat)	57.02	
Long*lat + T_ZMAX_	58.99	.88
Long*lat + T_TSB_	55.89	.09
Long*lat + D_ZMAX_	59.05	.96
Long*lat + C_ZMAX_	58.69	.45

Abbreviations: *C*_ZMAX_, maximum concentration of zooplankton in the water column; *D*_ZMAX_, depth of zooplankton maximum; long/lat, longitude and latitude coordinates where the sampling collection is located; *T*_SBT_, temperature at the sea bottom; *T*_ZMAX_, temperature at depth of zooplankton maximum.

## 4 DISCUSSION

Using SNP markers developed de novo for the current analyses, we here present the first population genetic data for the North Sea keystone species lesser sandeel. Our analysis returned two main results (a) lack of geographically explicit structure across the majority of examined loci and (b) the existence of genetic structure separating individuals into three distinct groups, mainly driven by 13 SNPs in relatively strong linkage disequilibrium (LD).

### 4.1 A putative origin of the sandeel clusters

In combination, the existence of three discrete clusters due to the presence of SNPs under strong LD suggests the presence of a genomic region with suppressed recombination that maintains divergent haplotypes within the populations of sandeel. The suppression of recombination could be linked to the centromere of the chromosome (Gagnaire et al., 2018; Roesti, Moser, & Berner, 2013) or to the presence of structural variants (SVs) in the sandeel genome (Wellenreuther, Mérot, Berdan, & Bernatchez, 2019), and it can be difficult to distinguish between the two in the absence of detailed genomic information. When a SV appears in the genome, the new variant is commonly described as the “derived” haplotype, where the “ancestral” haplotype would carry most of the genetic variation (Butlin, 2005; Kirkpatrick, 2010). For a diploid organism such as the sandeel, the three PCA clusters (Figure 1b) seemingly correspond to the three possible karyotypes of a SV, as observed for SVs in other organisms (e.g. Gazave et al., 2016; Ma & Amos, 2012). The homokaryotype individuals carrying two copies of the same haplotype would correspond to clusters 1 and 3 localized on the extremes of axis 1 in the PCA, and the heterokaryotype individuals carrying one copy of each derived and ancestral haplotype would correspond to admixed individuals localized in the centre of the PCA (cluster 2). The ADMIXTURE analysis further corroborated that cluster 2 consisted of admixed individuals from clusters 1 and 3 (Figure 1c) that exhibit a high level of heterozygosity (0.71), as expected from heterokaryotype individuals carrying one copy of each of two divergent haplotypes. The important divergence of the haplotype was also confirmed by the differentiation estimated between the two homokaryotype clusters (*F*_ST_ cluster 1 vs. cluster 3) which was several orders of magnitude higher with the 13 loci under LD (*F*_ST_ = 0.77) than estimates across all other loci (*F*_ST_ = 0.003). Both LD and *F*_ST_ estimates for the 13 SNPs are close to the values of LD and *F*_ST_ value from genomic regions containing SVs in other marine fishes in the same area (e.g. Berg et al., 2016 for Atlantic cod, and Le Moan, Bekkevold, & Hemmer‐Hansen, 2019 for European plaice).

Two types of SVs can result in these patterns of clustering observed in Figures 1b and 2a, that is inversions and translocations. It is not unusual that species carry more than one SV in their genomes, for example five inversions are reported in Atlantic cod (Wellenreuther & Bernatchez, 2018) and two in European plaice (Le Moan, Bekkevold, et al., 2019). The pattern of discreet clustering for PC2 of the 13 most linked loci (Figure 2a) might reveal a second SV, where the observed 9 clusters could correspond to the 9 genotypes expected for two SVs when not in full LD. Our data did not allow us to estimate the age or size of the potential SVs. RAD‐sequencing genotyping, the approach used in our study, is generally known to be biased towards the identification of large SVs (Wellenreuther & Bernatchez, 2018). In our study, 1% of the SNPs had high values of LD, and 13 of those (0.5% of our markers) were found in the LDna analysis and interpreted here to represent SVs. The genome size is unknown for *Ammodytes* spp. and relatives, but if we assume sandeel to have a typical fish genome size (~600 Mbp), the hypothesized SVs would be ~3 Mb. The average distance between the closest and furthest high‐LD SNP that blasted within the same fish species was ~5 Mbp (Table S3). Together, these two rough estimates suggest that the lesser sandeel SV size is within the broad size range of reported inversions (from 130 kbp to 100 Mbp, see Wellenreuther & Bernatchez, 2018). Hence, collectively, our data are consistent with the presence of major SVs in the sandeel genome.

The growing number of studies reporting the presence of SVs shows that SVs may be more widespread than it was originally thought (Wellenreuther & Bernatchez, 2018). Several evolutionary mechanisms can be responsible for the origin of SV polymorphism within a population (reviewed in Wellenreuther & Bernatchez, 2018 and Faria, Johannesson, Butlin, & Westram, 2019). For instance, SVs can appear following a period of gene flow during reticulate evolution (introgressive hybridization, see, e.g., Mavárez et al. (2006) or secondary contact, McGaugh and Noor (2012)) or simply arise de novo in the populations under study. Maintenance of the SVs in the populations may involve balancing selection including heterosis (Hoffmann, Sgrò, & Weeks, 2004), trade‐off between different life history traits (Mérot et al., 2018) and adaptation to micro‐habitat (Johannesson et al., 2010). Our study supports data from a number of other marine fish species in the North Atlantic (Berg et al., 2017—Atlantic cod; Le Moan, Bekkevold, et al., 2019—European plaice, Pettersson et al., 2019—Atlantic herring) and suggests that SVs may represent a significant part on intra‐specific genetic variation in these species. It is a currently unknown if the putative SVs in lesser sandeel originated in the populations presently inhabiting the North Sea or through gene flow from other populations or species. Future work could include wider geographical sampling within the Atlantic to obtain a better understanding of the SV distribution and origin in this species. Although not statistically significant, our analyses revealed a weak association between sea bottom temperature and SV haplotype frequencies, suggesting a possible role for selection in maintaining SV polymorphisms in the species. Given that sandeel spp. seem to have different habitat preferences (Endo, Iwasaki, Shibata, Tomiyama, & Sakai, 2019; Wright et al., 2000), the SVs could be related to adaptation to different micro‐habitats within the North Sea (e.g. see Van Belleghem et al., 2018). Also, timing and duration of the annual feeding window have been proposed as a potential driver of live history adaptation (van Deurs, Christensen, Frisk, & Mosegaard, 2010). However, the functional implications of the different genotypes are so far unknown, and further studies should include individuals from additional areas and different subhabitats to identify potential links between SVs and adaptive and demographic processes. Additionally, incorporation of age‐segregated data into the analysis might add further insight into relationships between genotypes and environmental factors. Finally, increasing genomic resolution could improve our understanding of both origin and evolution of SVs in the sandeel genome. Nevertheless, the putative sandeel SVs present exciting perspectives for an assessment of genetic connectivity in this species.

### 4.2 Implications for management

While panmixia was inferred from the total SNP data set, analyses of haplotype frequencies of the putative SVs revealed more fine‐scale population structure within the North Sea with potential implications for fisheries management. We found that the area SA4 was the most divergent of all. Particularly, the sample WB16 differed strongly from all other collections. When SA4 samples were excluded from analysis, the variation explained by management areas decreased to statistical nonsignificance, although the easternmost sample in the analysis, from SA2, also showed differentiation from most other collections in the pairwise *F*_ST_ comparisons. Our genetic results are hence in line with conclusions from Wright, Régnier, Gibb, Augley, and Devalla (2018) and Wright et al. (2019) who used inference from biophysical model simulations of larval transport and otolith chemistry to examine lesser sandeel connectivity within the North Sea. They found that both types of analyses supported that isolating mechanisms (i.e. limiting dispersal between certain areas) may act on local to regional scale which could lead to some reproductive isolation among subcomponents. Interestingly, they identified relatively stronger biophysical isolation of sandbanks located in SA4 than among other management subareas. Although inference to some extent hinges on a small number of high‐LD loci, our results are in line with this finding, as they showed indications of relatively larger reproductive isolation between SA4 and the rest of the management areas included in the study.

In Atlantic cod, coastal and migratory ecotypes are closely associated with the presence of large SVs (Berg et al., 2017) and are managed separately based on genotyping of representative samples collected on fishing grounds where the distribution of the two ecotypes overlaps (Dahle et al., 2018). In principle, it is possible that the putative sandeel SVs are linked to ecotypes, which should then consequently, and ideally, be managed separately. However, in the case of sandeel, heterozygous individuals are found in high frequency throughout collection sites across the North Sea, occurring in frequencies expected under Hardy–Weinberg equilibrium (Table S7). This suggests that all haplotypes belong to the same population and cannot be considered putative ecotypes. In contrast, we find that our data are more consistent with genetic variation segregating within and among populations, potentially under local selection, as discussed above.

Among the remaining management areas, we found that samples aggregated by fisheries management area did not show higher overall differentiation than individual collections within management areas, suggesting that current management units are not generally associated with diverging genetic profiles. However, the determination of dynamics within and among potential subpopulations in these management areas requires additional analysis as our results are based on a relatively limited number of samples with restricted temporal resolution. It should also be noted that demographic diversity of major importance to fisheries management may not have been reflected in our genetic data, as fisheries management typically operates under the ecological population paradigm while our data mainly reflect evolutionary processes over longer timescales (Waples & Gaggiotti, 2006).

In the light of the prediction that changing climatic conditions may affect North Sea sandeel productivity negatively (Lindegren et al., 2018), it would be of importance for the ecosystem approach to fisheries management to describe and monitor dynamics of subunits with different adaptive potential, for example linked to SVs, to avoid depletion of biodiversity that could potentially lead to population decline (Reiss, Hoarau, Dickey‐Collas, & Wolff, 2009). Further development of genetic resources is needed to accomplish this, that is to improve genomic characterization of the genetic variants we hypothesize to represent SVs with potential adaptive significance. This knowledge could be further implemented into a genetic tool that can be applied to monitor populations in time and space (Dahle et al., 2018; Hemmer‐Hansen et al., 2019; Nielsen et al., 2012).

Finally, we highlight the importance of using large numbers of markers distributed across the genome to fully characterize the genetic diversity of species and populations. In our case, this allowed us to detect subtle differentiation that otherwise could have been overlooked. This is particularly challenging in species with high gene flow, such as many marine fishes, where low levels of genetic differences across most of the genome can mask genetic divergence of strong functional significance. Thus, our study also serves as an example of the increased power offered by population genomics for conservation and management (e.g. Allendorf, Hohenlohe, & Luikart, 2010; Benestan et al., 2016; Hunter, Hoban, Bruford, Segelbacher, & Bernatchez, 2018).

## CONFLICT OF INTEREST

None declared.

## Data Availability

Data available from the Zenodo Digital Repository: https://doi.org/10.5281/zenodo.3458888. (Jiménez‐Mena et al., 2019)
