# Supplementary material for: Weak genetic structure despite strong genomic signal in lesser sandeel in the North Sea
Source: Evol Appl. 2019 Nov 1;13(2):376–87. doi: 10.1111/eva.12875 (PMC6976957; doi:10.1111/eva.12875)
Supplement: Supplementary file 3 [file EVA-13-376-s003.pdf]

Supplementary material for article

"Weak genetic structure despite strong genomic signal in lesser sandeel in the North Sea" by Jiménez-Mena *et al*.

Location (ICES management area)	Collection name	Collection ID	Sampling date	Latitude start (decimal)	Longitude start (decimal)	No. of samples taken at sampling	No. of samples sequenced	No. of samples after filtering
North Sea (SA 1)	Dogger West (Rute 18)	DW15	28.11.2015	54.99158 N	1.84315 E	50	44	40
	Dogger West (NW Rough)	DW16	27.11.2016	54.84616 N	1.43191 E	96	44	44
	Dogger South (Outer Well Bank)	DS15	25.11.2015	54.15220 N	2.06380 E	50	44	43
	Dogger South (SW Spit)	DS16	25.11.2016	54.18695 N	1.58111 E	117	44	43
	Dogger East (Elbov Spit)	DE16	23.11.2016	55.41518 N	4.31126 E	61	44	44
North Sea (SA 2r)	Vestbanke	VB 16	04.12.2016	57.04328 N	6.99681 E	115	44	34
	Det Jævne	DJ16	06.12.2016	57.40231 N	8.63913 E	69	44	43
North Sea (SA 3r)	Outer Shoal	OS15	03.12.2015	57.27380 N	5.09026 E	50	42	18
	Oestbanke	OB16	29.11.2016	57.75391 N	3.67653 E	50	42	32
North Sea (SA 4)	Turbot Bank	TB16	16.12.2016	57.42726 N	1.11908 W	70	44	38
	Wee Bankie	WB16	12.12.2016	56.22783 N	2.06100 W	70	44	37
Norwegian Sea	Runde	Ru16	01.07.2016	62.74100 N	5.62100 E	100	40	13

Table S1: information about the sampling of the individuals, including geographical locations (collections) and dates. The number of individuals that passed the filtering steps as described in the main article is also included.

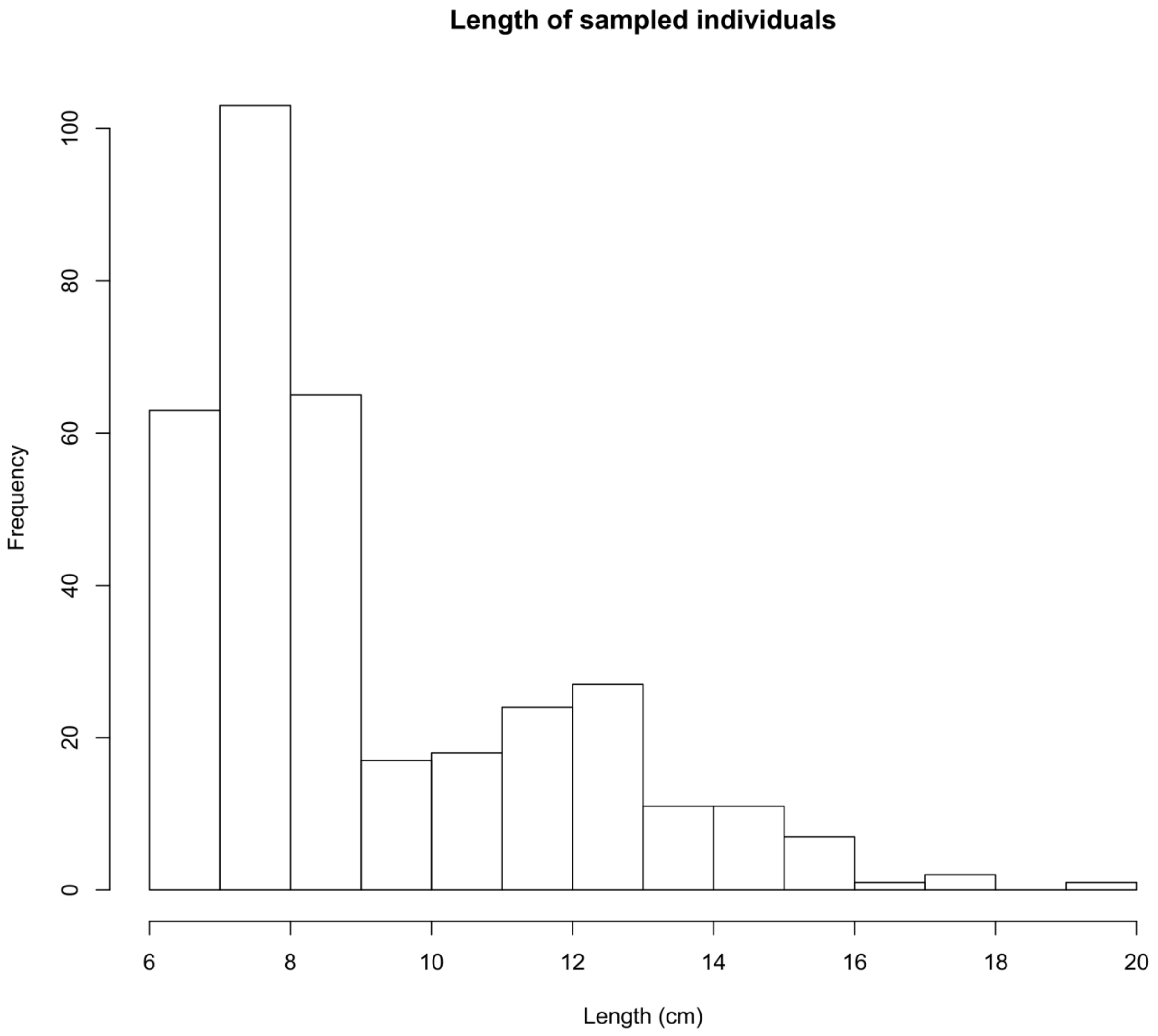

Figure S1: distribution of length of the sampled individuals.

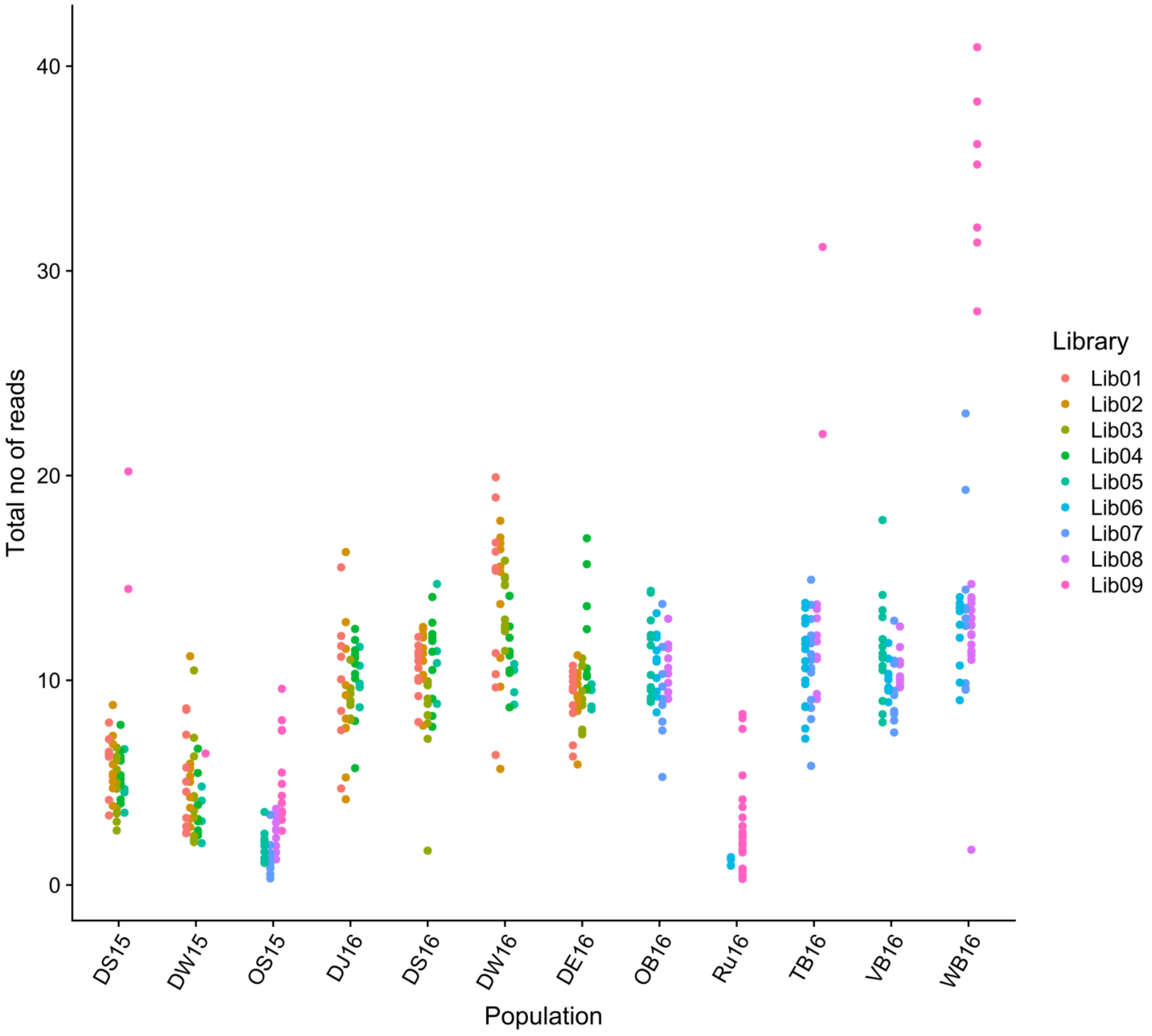

Figure S2: distribution of the total number of reads per sampling collection. Each dot represents an individual. Colours indicate the High-Sequencing library lane in which each individual was sequenced. Collections are named according to their location ID, indicated in Table S1.

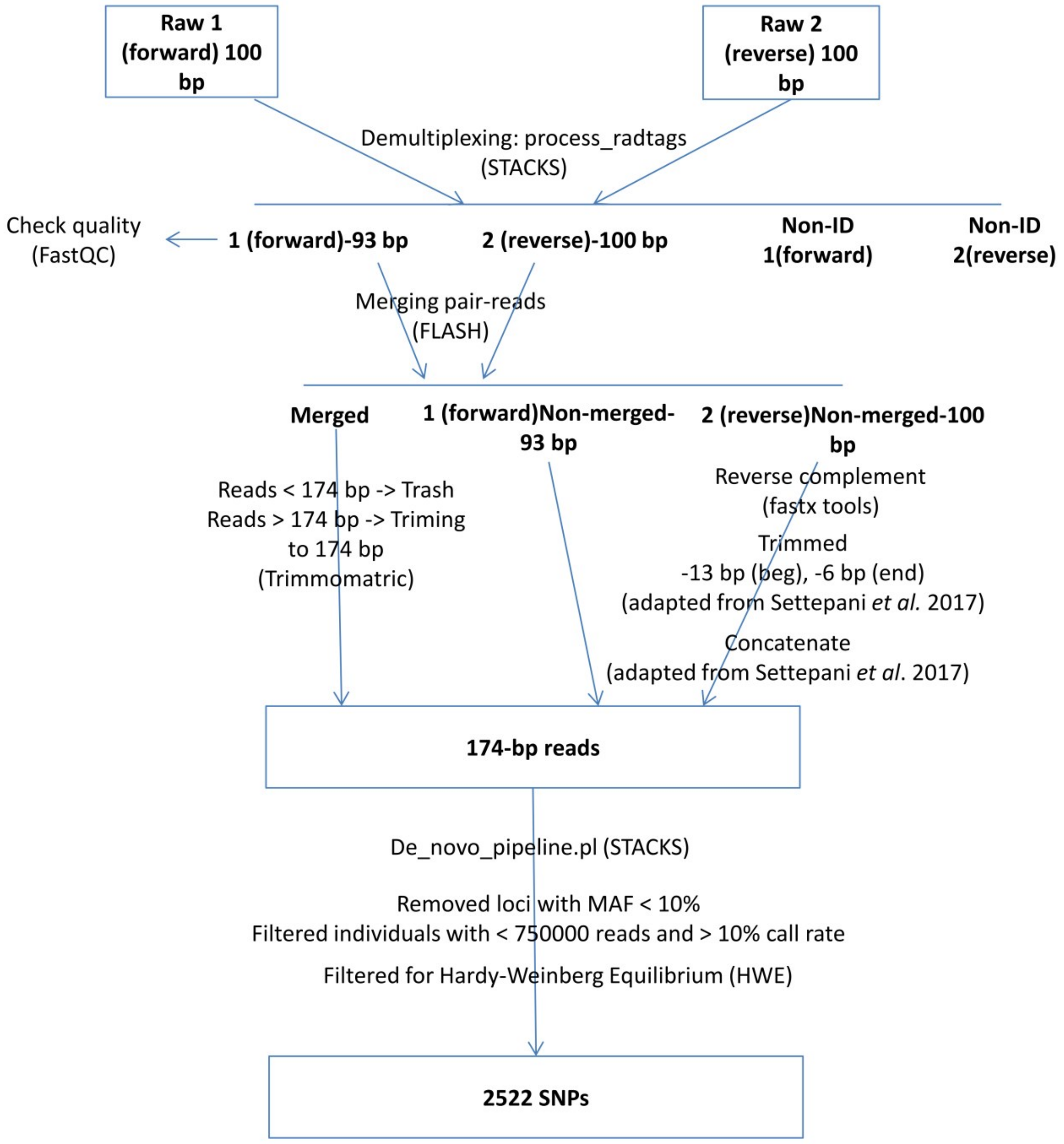

Adaptors = 7 bp

Figure S3: pipeline scheme of the main bioinformatics steps carried out for the analysis.

	DS15	DW15	DJ16	DS16	DW16	DE16	OS15	OB16	VB16	TB16	WB16	Ru16
DS15	NA	0.0109	0.0259*	-0.0073	0.0017	-0.0076	0.0057	-0.0091	-0.0080	-0.0007	0.0476***	-0.0048
DW15	0.0004	NA	-0.0082	0.0041	0.0172	0.0107	-0.0068	0.0052	0.0011	0.0404*	0.1127***	0.0350
DJ16	0.0004	0.0003	NA	0.0161	0.0374*	0.0279*	-0.0027	0.0222*	0.0137	0.0625***	0.1449***	0.0659*
DS16	-0.0003	0.0008	-0.0002	NA	0.0003	-0.0057	-0.0029	-0.0090	-0.0093	0.0030	0.0569***	0.0006
DW16	0.0004	0.0015	0.0011	0.0003	NA	-0.0027	0.0212	-0.0027	-0.0060	0.0009	0.0322***	-0.0121
DE16	0.0008	0.0006	0.0009	0.0003	0.0000	NA	0.0025	-0.0107	-0.0054	0.0005	0.0421***	-0.0107
OS15	0.0005	0.0000	0.0001	-0.0001	0.0014	0.0004	NA	-0.0034	0.0001	0.0321	0.1047***	0.0370
OB16	-0.0002	0.0003	0.0015	0.0006	0.0002	0.0004	-0.0015	NA	-0.0116	0.0042	0.0527***	-0.0055
VB16	-0.0005	-0.0015	0.0002	-0.0009	0.0000	-0.0004	-0.0010	-0.0016	NA	0.0024	0.0513***	-0.0037
TB16	0.0009	0.0012	0.0016	0.0003	0.0003	0.00071	0.0002	0.0002	-0.0004	NA	0.0121	-0.0165
WB16	0.0011	0.0029	0.0036*	0.0011	0.0013	0.001	0.0031	0.0011	0.0005	-0.0002	NA	-0.0038
Ru16	0.0001	0.0004	0.0013	0.0005	-0.0007	-0.0006	-0.0004	-0.0014	-0.0016	-0.0008	0.0007	NA

Table S2: Pairwise F_{ST} (Weir and Cockerman 1984) between the different collections: below diagonal with all the SNP dataset (2522 SNPs), above diagonal: only including the 13 most-linked SNPs identified in *LDna* (cluster “49_0.18”). Statistical significance after Bonferroni correction is reported as: * p-value ≤ .05; ** p-value ≤ .01; *** p-value ≤ .001

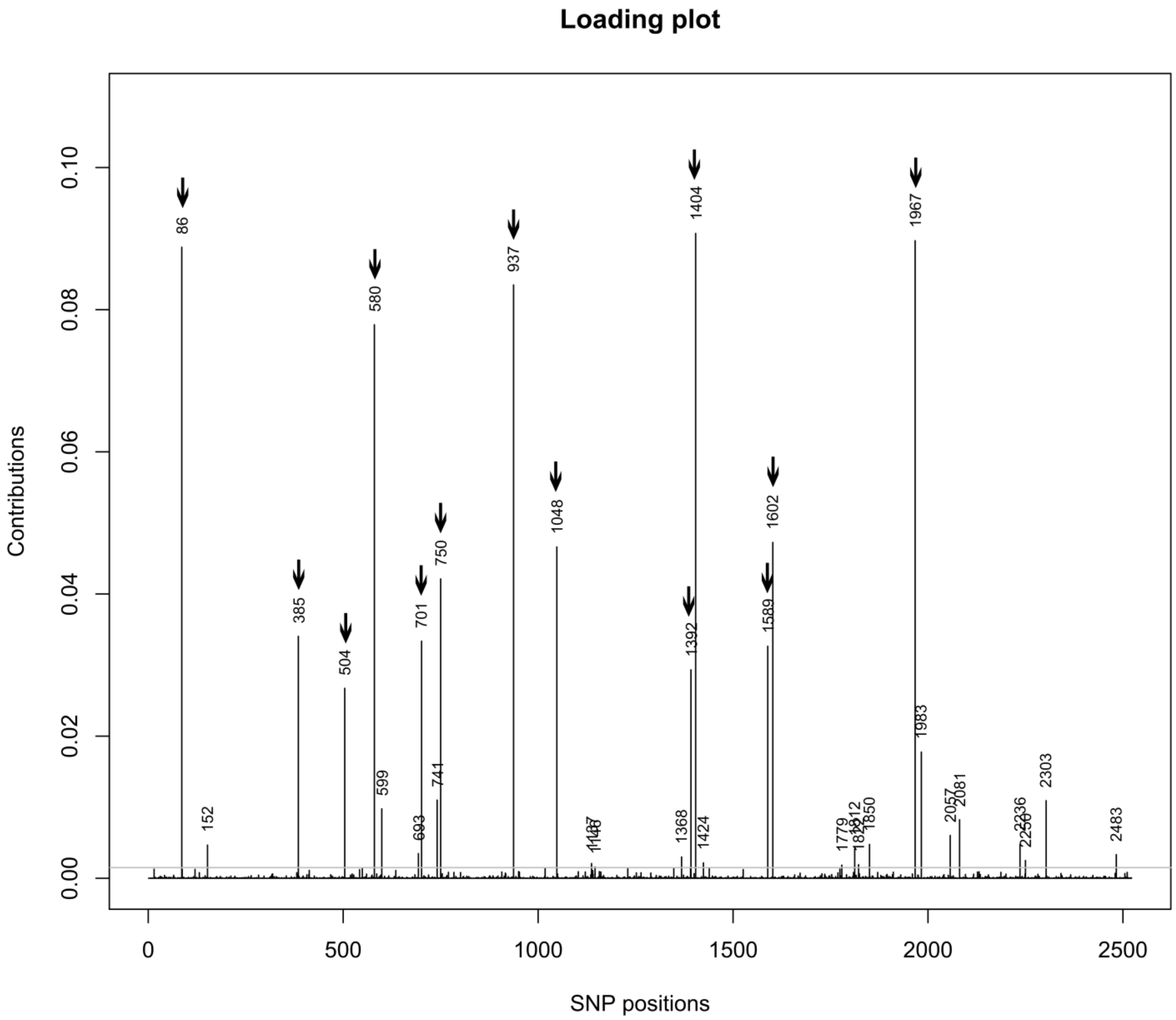

Figure S4: Loading plot of SNPs contributions to the axis 1 of the PCA in Figure 1B. The grey dashed line indicates the 95% quantile. Only the index IDs from the SNPs above the quantile are included. Arrows on top of the index IDs indicate the 13 most-linked SNPs identified in *LDna* (cluster “49_0.18”). SNPs with IDs 86, 580, 937, 1048, 1404, 1602 and 1967 are also included within the 99% of the quantile.

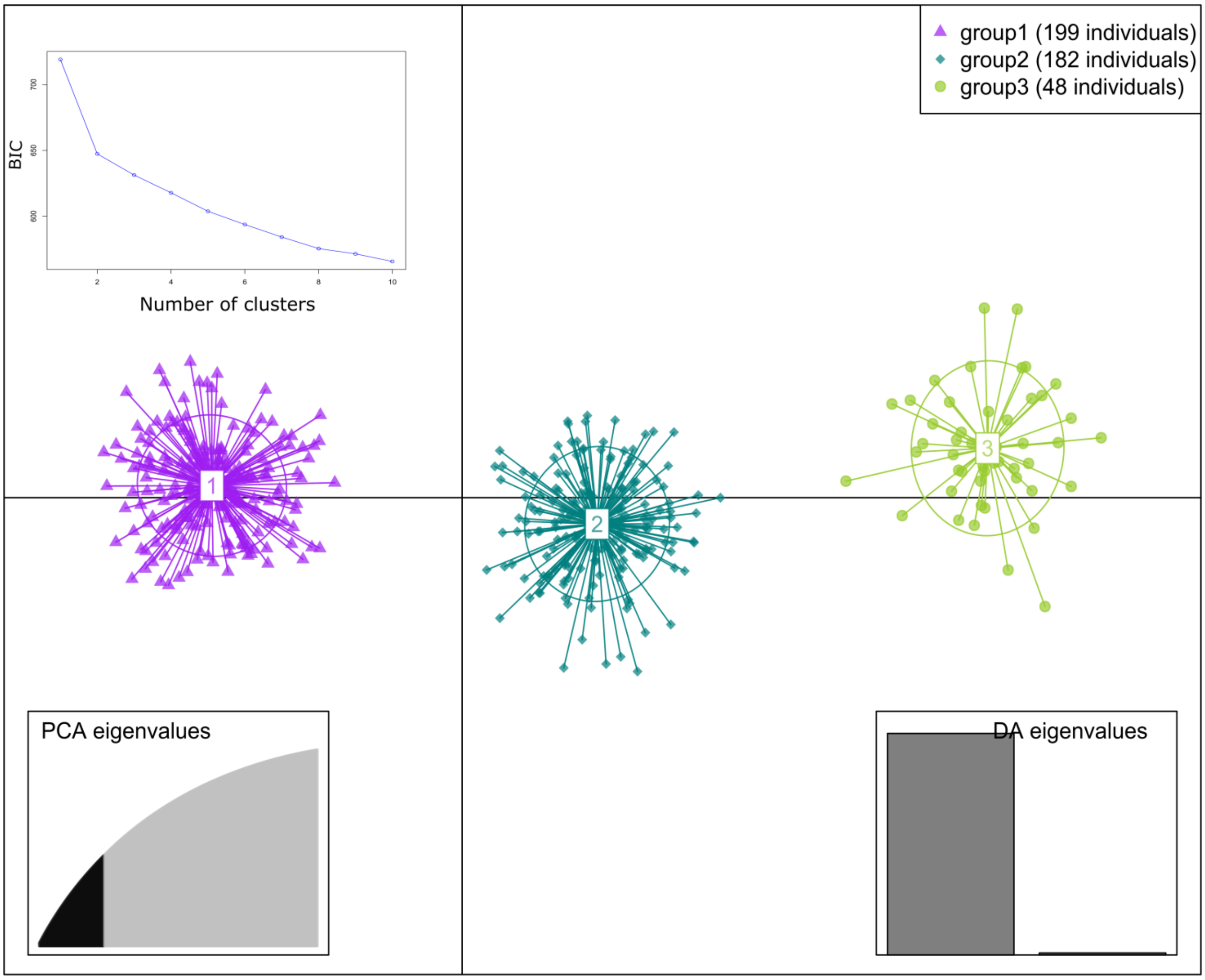

Figure S5: Results from the Discriminant Analysis of Principal Components (DAPC) for the panel of 2522 SNPs. Scatterplot representing the 3 genetic groups identified by DAPC, using 100 principal components and 2 discriminant functions. Individuals are represented by dots and population by circles. The number of individuals per identified cluster group are included in the legend. Upper left inset: visualization of the number of clusters that are identified following the Bayesian Information Criterion method implemented in the DACP method. For visualization purposes and the prior knowledge from Admixture, k=3 are selected.

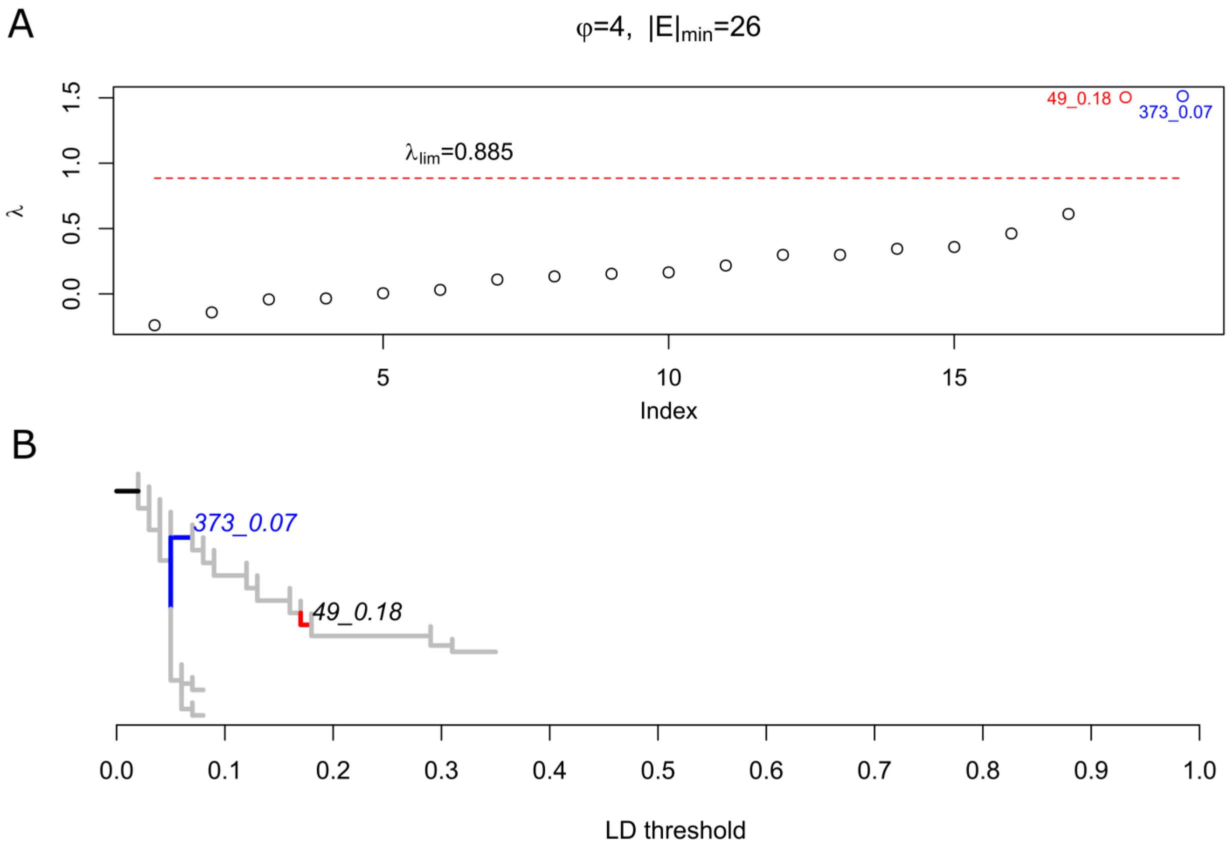

Figure S6: Results from the *LDna* analysis with a $|E|min = 26$ (minimum of loci to be considered an outlier cluster) and $\varphi = 4$. A) plot of "lambda" values, which represent the change in the median LD between all loci in a cluster before and after merger; B) tree summary of the SNP clustering based on the LD threshold obtained with the function *extractClusters()* of the *LDna* analysis. Cluster "49_0.18" merges at 0.17, referring to the LD value where the cluster unites with the next cluster.

Table S3: Excel file with the DNA sequences and BLAST results from (1) the SNPs identified in the *LDna* analysis (cluster "49_0.18"), with its ID as created by STACKS and the sequence. (2) the outlier SNPs above 5% loading quantile of PC1 in Figure 1B.

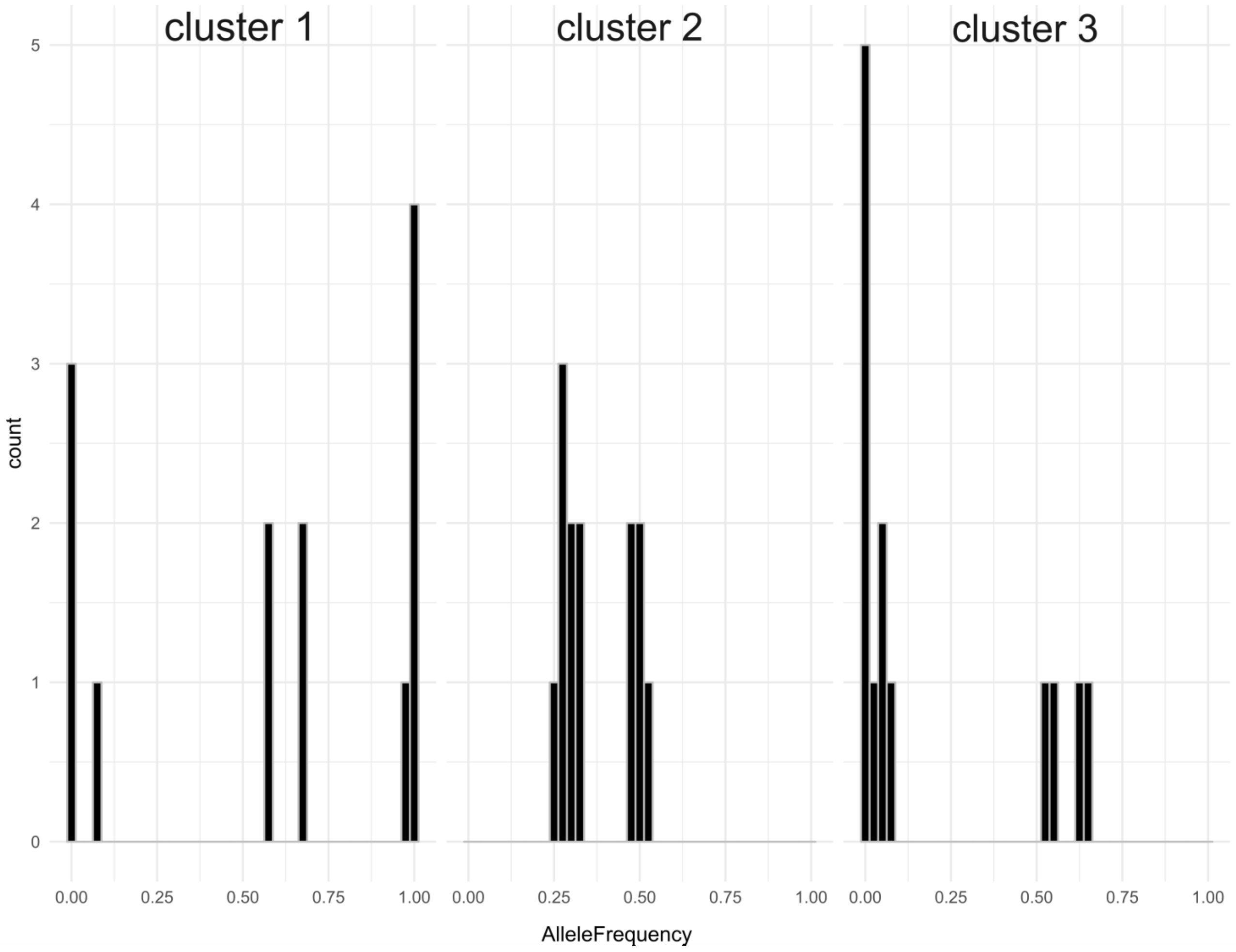

Figure S7: Histogram of the allele frequency of the 13 SNPs belonging to the group identified by *LDna* (cluster "49_0.18") with the highest LD, for each of the three clusters identified in Figure 1B and 2A.

Table S4: Excel file with the environmental factor values for all the collections. Columns represent the (1) year, (2) day, (3) collection ID number (1: DW15; 2: DW16; 3: DS15; 4: DS16; 5: DE16; 6: VB16; 7: DJ16; 8: OS15; 9: OB16; 10: TB16; 11: WB16), (3) T_{ZMAX} (temperature at depth of zooplankton maximum), (4) T_{SBT} (bottom temperature), (5) D_{ZMAX} (depth of zooplankton maximum), (6) C_{ZMAX} (zooplankton maximum concentration in the water column).

	Estimate	Std. Error	z value	Pr(>|z|)
(Intercept)	-3.94268	10.04773	-0.392	0.6948
Latitude	0.05502	0.17196	0.320	0.7490
Longitude	0.83473	3.38857	0.246	0.8054
T_{TSB}	0.14851	0.08916	1.666	0.0958
Latitude:Longitude	-0.01295	0.05956	-0.217	0.8279

Table S5: Coefficients from the *glm* test for the environmental factor (temperature at sea bottom, T_{TSB}) that showed a marginally significant correlation with allele frequencies of the *LDna* SNP group (group “49_0.18”).

Collection: TO<br>FROM	DW16	DS16	DE16	VB16	OS15	OB16	TB16	WB16
DW16	0.00744	0.00058	0.00000	0.00000	0.00000	0.00000	0.00000	0.00000
DS16	0.00560	0.01220	0.00000	0.00000	0.00000	0.00000	0.00000	0.00000
DE16	0.00000	0.00000	0.00626	0.00000	0.00000	0.00000	0.00000	0.00000
VB16	0.00000	0.00000	0.00044	0.00152	0.00254	0.00081	0.00000	0.00000
OS15	0.00000	0.00000	0.00000	0.00000	0.00018	0.00385	0.00000	0.00000
OB16	0.00000	0.00000	0.00000	0.00086	0.00187	0.01370	0.00000	0.00000
TB16	0.00000	0.00000	0.00000	0.00000	0.00000	0.00000	0.00025	0.00000
WB16	0.00000	0.00000	0.00000	0.00000	0.00000	0.00000	0.00315	0.01069

Table S6: Direct drift connectivity between the collections. As the connectivity matrix is uni-directional, the collections in rows (first column) represent the “donors” towards the collections in the columns (“recipient”).

ICES management area	Collection	χ^2 Statistic	P-value	χ^2 Statistic	P-value
SA1	DW15	1.27	1	0.25	1
	DS15	0.15	1		
	DW16	1.40	1	0.82	1
	DS16	0.45	1		
	DE16	1.15	1		
SA2r	VB16	3.66	0.67	4.12	0.7
	DJ16	0.26	1		
SA3r	OS15	0.23	1	0.94	1
	OB16	0.52	1		
SA4	TB16	4.98	0.31	6.12	0.22
	WB16	2.25	1		
Norwegian Sea	Ru16	0.58	1	-	-

Table S7: Results of the Chi-squared test for departures of Hardy-Weinberg equilibrium (HWE) within each collection, and within each management area, for the frequencies of the 13 most-linked loci highlighted by *LDna* (group “49_0.18”). P-values are represented after applying Bonferroni correction.
